# Shadow of a Pandemic: Persistence of Prenatal SARS-CoV-2 Antibodies in Newborn Blood Spots

**DOI:** 10.3390/ijns9030043

**Published:** 2023-08-02

**Authors:** Stanley Sciortino, Steve Graham, Toki Fillman, Hari Kandasamy, Robin Cooley, Carl Hanson, Valorie Eckert, Hao Tang, Juan Yang, David Seftel, Cheng-ting Tsai, Peter Robinson

**Affiliations:** 1 Genetic Disease Screening Program, California Department of Public Health, Richmond, CA 94804, USAhari.haran@cdph.ca.gov (H.K.);; 2 Viral and Rickettsial Disease Laboratory, California Department of Public Health, Richmond, CA 94804, USA; 3 Enable Biosciences, South San Francisco, CA 94080, USA

**Keywords:** antibody testing, COVID-19, dried blood spots, newborn, newborn screening, positive predictive value, pregnancy, prenatal, SARS-CoV-2, seroprevalence, serosurveillance

## Abstract

To investigate COVID-19 surveillance among pregnant women, the California Genetic Disease Screening Program conducted a screening performance and seroprevalence evaluation of maternal SARS-CoV-2 antibodies detected in banked newborn dried blood spots (DBS). We obtained seropositive results for 2890 newborn DBS from cohorts in 2020 and 2021 using Enable Bioscience’s Antibody Detection by Agglutination-PCR (ADAP) assay for SARS-CoV-2 antibodies. To infer maternal infection, we linked 312 women with a known laboratory-confirmed COVID-19 episode with their newborn’s DBS SARS-CoV02 antibody result. Among 2890 newborns, we detected 453 (15.7%) with SARS-CoV-2 antibodies in their DBS. Monthly snapshot statewide seroprevalence among neonates was 12.2% (95% CI 10.3–14.1%, *n* =1156) in December 2020 and 33.3% (95% CI 29.1–37.4%, *n* = 26) in March 2021. The longest time recorded from COVID-19 infection to a seropositive neonatal result was 11.7 months among the 312 mothers who had an available SARS-CoV-2 PCR test result. Approximately 94% (153/163) of DBS were seropositive when a known maternal infection occurred earlier than 19 days before birth. The estimated relative sensitivity of DBS to identify prevalent maternal infection was 85.1%, specificity 98.5% and PPV 99.2% (*n* = 312); the sensitivity was lowest during the December 2021 surge when many infections occurred within 19 days of birth. Fifty pre-pandemic specimens (100% seronegative) and 23 twin-pair results (100% concordant) support an intrinsic specificity and PPV of ADAP approaching 100%. Maternal infection surveillance is limited by a time lag prior to delivery, especially during pandemic surges.

## 1. Introduction

Pregnant women may be at increased risk for severe COVID-19-related illness, hospitalizations, and death compared to non-pregnant women of similar age [1,2,3,4,5], and severe COVID-19-related illness has been associated with adverse neonatal outcomes [6,7,8,9]. Prior to birth, transplacental transfer of maternal IgG antibodies occurs. Newborn screening (NBS) residual dried blood spots (DBSs) have been used to detect antibodies against several viruses transferred transplacental [10,11,12], including SARS-COV-2 antibodies [13,14,15,16,17,18,19]. 

The California Department of Public Health’s (CDPH) Genetic Disease Screening Program (GDSP) COVID-19 Active Perinatal Response to Infection (CAPRI) project leveraged identification of SARS-CoV-2 antibodies in banked residual NBS DBS to infer the cumulative seropositivity for these infants’ mothers. Antibodies detected in residual NBS DBS may provide a ready proxy for the cumulative maternal experience of SARS-CoV-2 infection if SARS-CoV-2 antibodies undergo transplacental transfer, the testing methodology has a high positive predictive value (PPV) for seropositivity, and if the test is not cross-reactive with other seasonal coronaviruses. 

CAPRI’s goal was to demonstrate the ability of routinely collected newborn DBS to indicate SARS-CoV-2 infection among infants’ mothers prior to or during pregnancy. Our objectives were to: (a) evaluate the intrinsic performance of a laboratory assay to detect antibodies in banked newborn DBS and the relative performance of DBS results to infer maternal SARS-CoV-2 infection experience prior to delivery, (b) determine the range of months before birth that maternal antibodies can be detected reliably in an infant’s DBS, (c) identify seroprevalence rates estimated from DBS results, and (d) examine demographic and anthropometric differences between exposed and unexposed newborns.

## 2. Methods

### 2.1. Study Population

Newborn screening is mandatory and offered for approximately 1200 infants born each day in California [20]. From 12 through 72 h after birth, a neonate’s blood is collected on DBS filter paper, dried, and shipped for laboratory testing. Residual DBSs are banked by the California Biobank Program (CBP). Less than 1% of parents or guardians decline screening or opt out of specimen storage. GDSP also conducts prenatal screening (PNS) and stores a subset of second trimester blood specimens.

### 2.2. Maternal-Newborn Linkage

The California Reportable Disease Information Exchange (CalREDIE) includes all positive reverse transcription-polymerase chain reaction (RT-PCR) test results. 

We linked all female adolescents and women aged 12–55 years in California (1,312,600) who had a COVID-19 episode identified by CalREDIE to 444,700 pregnant women who had a live birth in California from 1 March 2020, to 31 March 2021, inclusive, using the following identifiers recorded by NBS, PNS, and Vital Statistics birth records: the maternal first, last, and maiden name, the infant’s date of birth, residential addresses and birth hospital. Linkage was independent of sample selection and laboratory testing.

The COVID-19 episode date is defined as the earliest date recorded in CalREDIE among the following: symptom onset, diagnosis, specimen collection, laboratory receipt, and CalREDIE record creation date.

CalREDIE episode days and months before birth were calculated as the maternal episode date minus the infant’s birthdate. We retained linkages that occurred both before and after birth for analysis. Negative episode dates occurred prior to birth and those identified at 0 days on the date of delivery were considered true-positive episodes relative to the newborn’s birthdate. Positive episode dates which occurred after the date of delivery were considered to be true-negative results relative to the newborn birthdate and we would expect no antibodies in the newborn’s DBS. 

We coded the self-reported race/ethnicity, multiple-choice response from our NBS test request forms as Hispanic of any race, then non-Hispanic single-race, then multiple races, and other (including unknown).

Maternal age in years at birth equaled the maternal birthdate minus the infant birthdate. Gestational age in weeks and birthweight in grams were taken from NBS records and used to ascertain if these measures differed among infants with and without SARS-CoV-2 antibodies.

### 2.3. Laboratory Testing

For all specimens, we employed Enable Bioscience’s Antibody Detection by Agglutination-PCR (ADAP) test for SARS-CoV-2 antibodies, described previously [21,22,23,24]. ADAP is pan-specific for any isotypes of SARS-CoV-2 antibodies (e.g., IgA, IgM, IgG). Unless specified otherwise, ADAP uses protein subunit 1 (S1)-DNA conjugates as probes for both NBS DBS and prenatal serum specimens. Laboratory results indicated the dimensionless signal of ADAP assay as the change in cycle threshold (ΔCt) as the cycle threshold (Ct) value difference between the sample and a blank control (ΔCt = Ct_blank_ − Ct_sample_). The assay cutoff was established as the 99th percentile of healthy controls’ DBS or serum sample ΔCt. 

To verify if an NBS result was due to natural infection for selected specimens, Enable deployed an assay for the presence of antibodies to nucleocapsid protein (NP) not stimulated by vaccination. Laboratory scientists were provided de-identified specimens and were blinded to linkages. 

### 2.4. Cohorts

October 2020 pilot: In October 2020, we initiated a pilot study to evaluate the CAPRI methodology. Statistical sampling strategies to identify prevalence rates lower than 10% can lead to missed true-positive results and may emphasize false-positive antibody test results [25]. To augment suspected low seroprevalence in October 2020, we chose residential zip codes suspected to be hotspots of SARS-CoV-2 infection and selected all DBSs of infants born in these areas to capture rare cases. Based on a demographic analysis of geographic data, we chose one zip code of residence at birth with the greatest number of births in each of 11 populous counties in California. We tested all newborn DBS accessioned during October 2020 in selected zip codes. These areas tended to be densely populated and demographically diverse but did not represent births statewide.

December 2020 snapshot: We selected a 1-day snapshot sample of the entire state in December 2020 of all NBS specimens accessioned midweek prior to initiation of COVID-19 vaccinations. Areas selected for October 2020 and specimens selected for December 2020 are shown on a supplemental map of the state (Figure S1).

March 2021 snapshot: We selected a simple random subsample of a snapshot accessioned on a single weekday.

Twins: We identified all twin pairs within our pandemic cohorts as an internal reference for concordance. One twin of each pair was randomly selected and retained for all other analyses that represent single pregnancies.

Pre-pandemic specimens: To estimate specificity intrinsic to ADAP, we chose pre-pandemic DBS specimens randomly from newborns screened in April 2019 at the end of the influenza and respiratory virus season in California to challenge ADAP with potential cross-contamination from other coronaviruses [26,27,28]. 

Prenatal specimens: We identified second trimester banked prenatal serum specimens within our cohorts collected shortly before or after a positive COVID-19-episode date and tested these for SARS-CoV-2 antibodies.

### 2.5. Statistics

We estimated a minimum sample size of 1188 to reliably detect 1% seroprevalence using the exact binomial distribution (Power = 0.95, 95% Confidence Interval (CI) 0.5–1.6%). The sample size coincided with the daily births and our mid-week laboratory testing volumes, which would be sufficient to conduct single-day snapshot estimates of statewide seroprevalence.

We evaluated twin concordance using the Cohen Kappa statistic and exact binomial probabilities. For pre-pandemic and twin results, we estimated single proportion specificity, sensitivity, and positive predictive value (PPV) using Jeffreys 95% credible intervals with SAS [29,30]. The CalREDIE mother–newborn pandemic cohorts were heterogeneous so we combined them for a relative performance analysis to estimate prenatal infection from DBS seropositivity using bivariate Bayes estimates in SAS NLMIXED models [31]. We simplified the Rogan–Gladen equation (RGE) by applying a test specificity of 100% to provide a crude estimate of maternal prevalence for DBS collected during a the surge in infections that began in November 2020 and peaked in late December by dividing prevalence by relative sensitivity for a given month [32,33]. 

For the demographic analyses, we calculated binomial confidence intervals, multivariate Poisson prevalence ratios, 2-sample t-tests, and created graphics using SAS ver. 9.4 (SAS Institute, Carey, NC, USA). Binomial power calculations were obtained using R (ver. 3.8.2). 

## 3. Results

We selected a total of 2963 DBS specimens, including 50 chosen before the pandemic (Table 1). Among these we identified 23 twin pairs and 2940 unique pregnancies. We matched the maternal cohorts with 312 laboratory-confirmed COVID-19 infections in CalREDIE, which were used for the relative performance analysis. We matched seven prenatal serum specimens available in our biobank collected before or shortly after their confirmed COVID-19 prenatal episodes; one of the women identified gave birth to a child with a DBS sample in our maternal cohort. 

**Figure 1:**
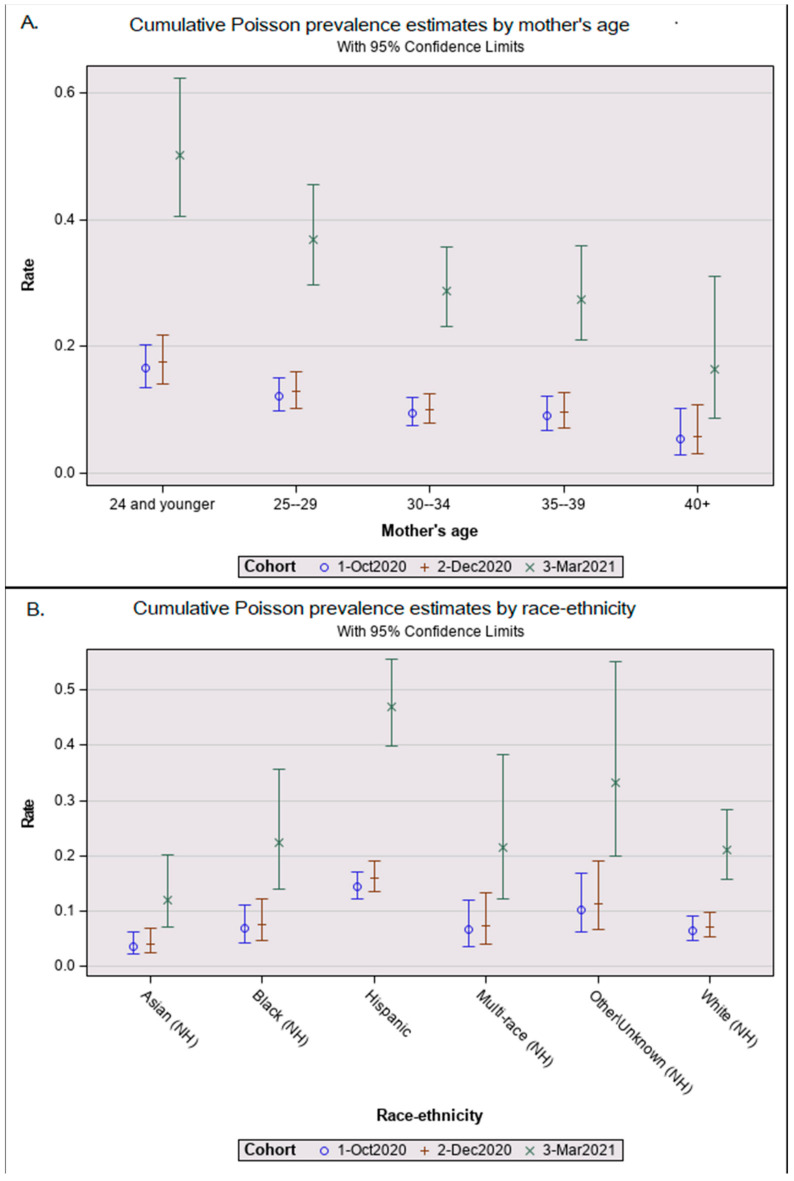
Adjusted Poisson prevalance estimates by cohort. Graphical results from a Poisson prevalence analysis showing whisker plots of least squares means as central circles and upper and lower 95% confidence interval bars for (**A**) maternal age group and (**B**) newborn race-ethnicity by the three cohorts: 1 October 2020 pilot, 2 December 2020 snapshot, and 3 March 2021 snapshot.

Table 2 showed the 453 seropositive results from infant DBSs yielding a seroprevalence of 11.9% in October 2020, 12.2% in December 2020, and 33.3% in March 2021. In the multivariate Poisson analysis in Table 2, seroprevalence was three times higher in March 2021 than in December 2020 after adjusting for maternal age at delivery and infant race/ethnicity (Table 2). The relationships between maternal age (Figure 1A) and infant race/ethnicity (Figure 1B) did not differ significantly among the cohorts; therefore, we combined all age- and ethnicity/race-adjusted results to simplify our demographic review. The seroprevalence among Hispanic infants was 2.1 (95% CI = 1.5–2.8; *p* < 0.001) times that of white infants. Non-Hispanic Asian babies had the lowest seroprevalence ratio of 0.6 (95% Poisson CI = 0.3–1.1; non-significant). Mothers aged 15–24 years had seroprevalence ratios 2.6 times (95% CI = 1.4–5.0; *p* < 0.01) higher than mothers aged 40 years or older (Table 2). The mean maternal age at birth also differed significantly between seropositive (mean = 28.1 years, SD = 5.9) and seronegative newborns (mean = 29.7, SD = 5.9, *t*(2,887), *p* < 0.001).

Birthweight did not differ significantly between seropositive (mean = 3283 g, SD = 552) and seronegative (mean = 3311 g, SD = 575, *t*(2,876) *p* = 0.32) newborns. Gestational age also did not differ significantly between seropositive (mean = 38.5 weeks, SD = 1.75) and seronegative newborns (mean = 38.6 weeks, SD = 1.81, *t*(2,879) *p* = 0.32). We further examined whether preterm births (<37 weeks gestational age) were associated with antibody status and found no statistically significant results in a Mantel Haenszel Chi-Square analysis stratified by maternal age < 25 years (antibody detected yes = 5.1%, vs. no = 7.9% preterm births, *p* < 0.264, OR = 0.63 95, % CI = 0.27, 1.43, *n* = 660) and age 25 years or greater (antibody detected yes = 10.6%, vs. no = 8.0% preterm births, *p* < 0.1220, OR = 1.37, 95% CI = 0.92, 2.03, *n* = 2220).

### 3.1. CalREDIE Mother–Infant Linkage

**Figure 2:**
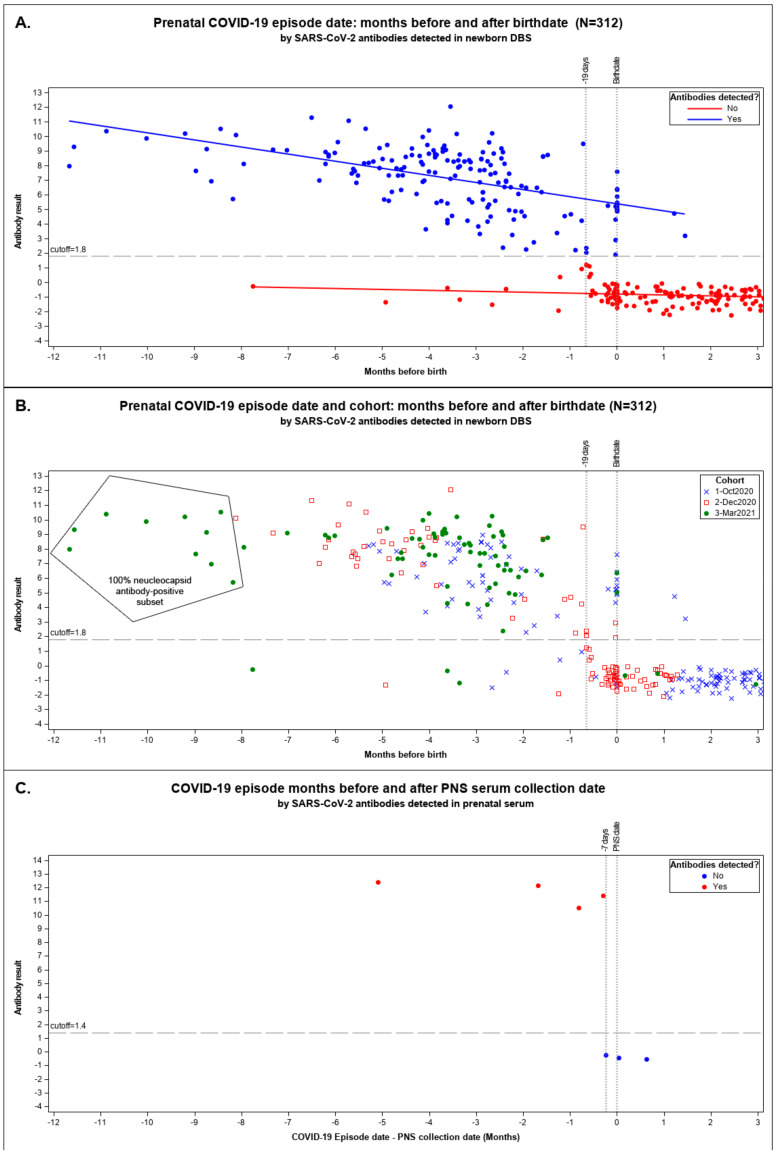
Maternal COVID-19 episode date: months before and after birth date. We plotted the months before and after birth of the mother–newborn matched data against the ADAP antibody results with all pandemic cohorts combined. Result indicates the dimensionless signal of ADAP assay ΔCt as the Ct value difference between the sample and a blank control (ΔCt = Ct_blank_ − Ct_sample_). The assay cutoff was established as the 99 percentile of healthy control’s dried blood spot ΔCt or serum sample. Scattergram (**A**) shows seropositive and seronegative results above and below the ΔCt result cutoff of 1.8. The linear regression lines were fitted to the following models: ADAP result(detected: Yes) = 6.049449 − 0.316978 * Months-before-birth, ADAP result(detected: No) = −0.796007 − 0.09239 * Months-before-birth. Scattergram (**B**) shows the same data identifying the cohort responsible for each data point: 1 October 2020 pilot, 2 December 2020 snapshot, and 3 March 2021 snapshot. NBS DBS from March 2021 found inside the polygon were tested for antibodies to nucleocapsid protein (NP); all with positive. No other DBS were tested for NP antibodies. (**C**) shows the months a COVID-19 episode was identified prior to prenatal specimen collection by SARS-CoV-2 antibodies detected in serum by ADAP. These 7 banked serum specimens where the only ones we could identify where the prenatal specimen was collected after or around the date of a known COVID-19 episode. The one prenatal serum specimen collected 5 months after an episode of COVID-19 was identified is the same pregnant woman linked to the DBS result listed in (**A**,**B**) over 9 months before delivery.

We plotted maternal COVID-19 episodes linked with their newborn’s seropositive results by months until birth for the pandemic cohorts in Figure 2A,B.

We observed a marked decline in the sensitivity of seropositive newborn DBS to infer seropositivity of the mother when the prenatal episode date occurred between 19 days prior to delivery and delivery (−19 to 0 days) inclusive (Figure 2A,B). Among CalREDIE cases, 93.9% reported prior to 19 days before birth matched with seropositive DBS and 97.9% dated after birth were seronegative DBS (Supplemental Table S1). Only 26.9% of mothers with episodes within 19 days of birthdate delivered babies with a seropositive result. 

The longest time to birth recorded for a mother–newborn seropositive result was 11.7 months (355 days) before delivery in March 2021 (Figure 2A,B). Since mothers may have been vaccinated in March 2021, we tested the persistence among the ten earliest recorded COVID-19 cases linked to DBSs from the March 2021 cohort with maternal cases dating over eight months prior to birth; all were NP-antibody-positive, suggestive of results due to natural infection [34]. 

Figure 2C shows the seven prenatal specimens coinciding with prenatal COVID-19-cases dated from 155 days before to 19 days after PNS serum collection. All four prenatal specimens collected more than 8 days after the episode date were antibody-positive. All three specimens collected from 7 days before to 20 days after an episode date had no detectable antibodies. One antibody-positive prenatal specimen indicated in Figure 2C was also linked to a seropositive newborn result in our study. That prenatal specimen was collected 155 days after the recorded episode date. No other prenatal specimens were available that linked to our newborns’ DBS. 

### 3.2. Screening Performance

#### 3.2.1. Intrinsic ADAP Performance

All 50 pre-pandemic samples were seronegative (specificity = 100%, 95% CI = 95.1–100%). All 23 twin pairs were concordant; five pairs were seropositive (sensitivity and PPV = 100%, 95% CI = 62.0–100%) and 18 pairs were seronegative. All seropositive pairs also had a mother with a confirmed CalREDIE case; no maternal cases were identified for seronegative twins. The twin–twin agreement yielded an exact Kappa statistic of 1 (*p* < 0001) and exact binomial probability <10^−5^ based on a seropositive probability of 0.22 (5/23). 

#### 3.2.2. Relative Performance

We pooled data from the three cohorts as if they were heterogeneous studies and estimated a combined relative sensitivity of 86.1% (95% CI = 79.3–91.0%), specificity of 98.1% (95% CI = 94.8–99.0%) and a positive predictive value (PPV) of 98.5% (95% CI = 94.6–99.6%) (Table 3). These pooled estimates account for contributions from the cohorts of varying prevalence and sensitivity; December 2020 had the lowest (55.7%) sensitivity of the three (October 2020 = 86.1%; March 2021 = 94.8%) due to the surge of acute infections occurring within 19 days of birth. 

We adjusted for the surge in December 2020 and March 2021 by dividing cohort prevalence by monthly relative sensitivity obtained from the linked CalREDIE data [32,33,35]: December 2020 yielded 22% (12%/56%) and March 2021 yielded 35% (33%/94%).

## 4. Discussion

In absence of a reference standard, our concordance and pre-pandemic results provided evidence for high intrinsic assay performance; each twin pair we identified was exposed to an identical uterine environment during gestation and each had concordant results, which were extremely unlikely to occur by chance; pre-pandemic specimens showed no false positive results. We could not rule out that sample degradation hampered detection of interfering coronavirus antibodies for the pre-pandemic specimens, but antibodies have been shown to be stable in newborn DBS specimens banked at −20 °C for up to 200 days [36]. We now use twin concordance to validate laboratory results from subsequent cohorts in an ongoing study.

PPV and specificity are crucial for surveillance; we need to be confident that a positive result is truly positive. The relative screening analysis reinforced our confidence in ADAP specificity and PPV, diverging from 100% due to two antibody-positive results when mothers were first identified with COVID-19 after delivery. The two false-positive results in Figure 2B were identified in the October 2020 cohort, which also had the most antibody-positive results identified at delivery suggestive of late identification of disease—a misclassification bias inherent in active testing of ongoing infection. Neither ADAP nor mother–newborn linked results are standards; either might be correct or incorrect. Pending clinical studies of ADAP, our results reinforce published reports of high ADAP performance [23,37] approaching 100% PPV and specificity sufficient for serosurveillance in DBS.

Our study identified a 19-day lag prior to delivery before antibodies were detectable in DBS and an analogous 7-day lag for a mother to produce detectable antibodies. The 19-day lag coincided with the time expected for a maternal humoral immunologic response to SARS-CoV-2 followed by transfer of IgG antibodies to the fetus [38] and detection of SARS-CoV-2 antibodies in the infant’s DBS. The few relative, false-negative mother–newborn results identified before this 19-day lag may be due to women whose maternal antibody titers decreased gradually after infection. Relative sensitivities in October 2020 (86.1%) and March 2021 (94.8%) were higher than that of December 2020 (55.7%), sampled during a pandemic surge in California [39] when many infections occurred within the 19-day lag period (Figure 2B). The surge adjustment we explored made a large difference in December 2020 (22% vs. 12%) but was not needed for March 2021 (35% vs. 33%), where relative sensitivity reflected limitations of antibody testing and not a surge.

Women who had confirmed COVID-19 cases before delivery transferred measurable antibodies to their newborns exceeding durations from studies that provided direct evidence of placental transfer up to 12 months post-infection [16,40,41]. To rule out confounding from vaccine-induced antibodies [42,43], we requested the ADAP test for the presence of antibodies to NP, post-hoc; all ten persistently seropositive mother–newborn results identified after eight months were NP antibody-positive (Table 2). We confirmed that the one woman who produced persistent antibodies also had seropositive prenatal serum collected five months after the episode date. Seroprevalence alone does not indicate the neutralizing activity of the antibodies present, which may provide limited protection against future viral variants and reinfection [44,45]. We do not know if any of the infants in our study acquired antibodies due to vertical infection before birth, but vertical transmission may be rare [46] and the presence of newborn antibodies still represents maternal exposure. Our results of antibody persistence may include instances of later maternal reinfection not captured by CalREDIE, and vaccine-induced antibodies in the March 2021 cohort. 

CAPRI reported that more SARS-CoV-2 antibodies were identified among Hispanic infants, supporting results from studies of adults [1,47], while Asian newborns had the lowest prevalence. Our use of race/ethnicity was intended to provide a social context to inform efforts to improve health equity among pregnant people and was not meant to imply inherent susceptibility. We observed that young women under 25 years of age were more likely to give birth to seropositive infants across all cohorts; young pregnant women remain vulnerable to SARS-CoV-2 infection and should be encouraged to vaccinate. 

Consistent with some other studies [4,5], gestational age at birth and birthweight were similar among seropositive and negative infants, indicating that maternal SARS-CoV-2 infection did not affect the basic health metrics of babies. Observations among live births may not capture other adverse outcomes of birth. However, our results did show greater odds (OR = 1.37) of preterm births (<37 weeks gestation) among women 25 years or older who gave birth to a seropositive newborn vs. a seronegative newborn, but the sample size was inadequate to detect a difference statistically. While there was no statistical association between antibody status and preterm birth among younger women, who constituted 23% of our sample, the effect was in the opposite direction (OR = 0.63) from older women. The results among women over 24 years, though not significant, do coincide with a recent study that showed an increased risk of preterm births among women who were infected with COVID-19 during their pregnancy [48]. That study had a smaller proportion of women younger than 25 years (12%) and did not stratify by maternal age. Since our specimens are available for testing and analysis, we may revisit this question during the entire course of the pandemic and see if this observation occurred by chance alone.

Prenatal administration of vaccines for SARS-CoV-2 is safe [49] and induces antibodies that are later transferred to the fetus [42,50]. Since March 2021, vaccination has been recommended for pregnant women; DBS testing can provide insight into infection and vaccination prevalence when coupled with data from CalREDIE and our statewide immunization registry. 

## 5. Conclusions

The CAPRI study provides evidence that maternally derived antibodies can be detected in newborn DBS for more than 11 months after maternal infection and throughout pregnancy until shortly before birth. California can leverage banked newborn DBSs for serosurveillance statewide to estimate prenatal infection among those who may not have access to SARS-CoV-2 molecular or antigen testing or who are asymptomatic and did not seek testing. Other states or countries do not need a biobank; they can sample the DBS at time of NBS to establish the infant’s serostatus shortly after birth. We found that ADAP may have high enough specificity and PPV to model infection dynamics among pregnant women from the beginning of the pandemic when prevalence was low, without interference from false-positive results.

## Figures and Tables

**Table 1:** California newborn blood spots selected and pregnancies used for screening analysis.

	All Cohorts			Relative Performance Analysis ^b^
Cohorts	Newborn Blood Spots Tested	Twin Pairs	Unique Pregnancies ^a^	CalREDIE Mother–Newborn Matches
Pre-pandemic	50	0	50	0
October 2020	1248	10	1238	131
December 2020	1167	11	1156	105
March 2021	498	2	496	76
Total	2963	23	2940	312

^a^ Unique pregnancies represent all cohorts including one twin of each twin pair selected randomly. ^b^ The relative performance analysis includes CalREDIE mother–newborn matches with a laboratory-confirmed episode of COVID-19 infection identified before and after birth and included the pre-pandemic cohort.

**Table 2:** California pandemic cohorts of newborn dried blood spot antibody results.

		N	Antibody Detected	Prevalence (95% CI) ^a^	Prevalence Ratio (95% CI) ^b^
	Total	2890	453	15.7% (14.3%, 17.0%)	
Cohort	October 2020	1238	147	11.9% (10.1%, 13.7%)	0.9 (0.7, 1.1)
	December 2020	1156	141	12.2% (10.3%, 14.1%)	1.0 ref
	March 2021	496	165	33.3% (29.1%, 37.4%)	3.4 (2.7, 4.2) ***
Race-ethnicity	Hispanic	1737	342	19.7% (17.8%, 21.6%)	2.1 (1.5, 2.8) ***
	Asian (NH)	270	15	5.6% (2.8%, 8.3%)	0.6 (0.3, 1.1)
	Black (NH)	204	19	9.3% (5.3%, 13.3%)	1.0 (0.6, 1.7)
	Multi-race (NH)	108	12	11.1% (5.2%, 17.0%)	1.0 (0.6, 1.9)
	Other (NH)	115	16	13.9% (7.6%, 20.2%)	1.6 (0.9, 2.7)
	White (NH)	456	49	10.7% (7.9%, 13.6%)	1.0 ref
Maternal age (years) ^c^	15–24	662	139	21.0% (17.9%, 24.1%)	2.6 (1.4, 5.0) **
	25–29	800	130	16.3% (13.7%, 18.8%)	2.0 (1.1, 3.9) *
	30–34	826	111	13.4% (11.1%, 15.8%)	1.8 (0.9, 3.4)
	35–39	471	63	13.4% (10.3%, 16.4%)	1.7 (0.9, 3.4)
	40+	130	10	7.7% (3.1%, 12.3%)	1.0 ref

Abbreviations: ref, reference; NH, non-Hispanic. ^a^ Confidence intervals based on unadjusted exact binomial estimates. ^b^ Prevalence ratios estimated by a Poisson analysis adjusted for race-ethnicity and maternal age. ^c^ Maternal age in years at delivery was missing in 1 case due to a lack of mother’s date of birth * *p*-values calculated by the multivariate Poisson prevalence analysis: * ≤ 0.05; ** ≤ 0.01; *** ≤ 0.001.

**Table 3:** Combined relative performance values based on parameter and empirical Bayes estimates for the mother–newborn linkage.

			Sensitivity		Specificity		PPV
Cohort	N	Observed ^a^	Estimated ^b^ (95% CI)	Observed ^a^	Estimated ^b^ (95% CI)	Observed ^a^	Estimated ^b^ (95% CI)
Combined ^c^	312	76.6%	86.3% (79.3%, 91.0%)	98.6%	98.1% (94.8%, 99.0%)	98.7%	98.5% (94.6%, 99.6%)
October 2020	131	85.9%	86.1% (75.8%, 92.5%)	97.0%	98.1% (94.3%, 99.4%)	96.5%	97.7% (83.6%, 99.7%)
December 2020	105	54.4%	55.7% (44.8%, 66.2%)	100.0%	98.5% (94.9%, 99.6%)	100.0%	99.1% (96.7%, 99.7%)
March 2021	76	95.8%	94.8% (87.8%, 97.9%)	100.0%	97.8% (94.2%, 99.2%)	100.0%	98.8% (95.1%, 99.7%)

Abbreviations: PPV, positive predictive value. ^a^ Unadjusted observed proportions. ^b^ Estimate proportions and confidence intervals using Bayes estimates. ^c^ October, December 2020 and March 2021 cohorts were treated as heterogeneous due the different sampling methodologies and prevalence. These were summarized using binomial Bayes analysis.

## Data Availability

Participant data cannot be made available due to legal and ethical requirements restricting access to individual level data from the California newborn screening program.

## Supplementary Materials

The following supporting information can be downloaded at: https://www.mdpi.com/article/10.3390/ijns9030043/s1.

## Abbreviations

ADAP	Antibody Detection by Agglutination-PCR assay
CalREDIE	California Reportable Disease Information Exchange
CBP	California Biobank Program
DBS	Dried blood spots
GDSP	Genetic Disease Screening Program
NBS	Newborn screening
PNS	Prenatal screening
PPV	Positive Predictive Value
RT-PCR	reverse-transcriptase PCR
