# Supplementary material for: Shadow of a Pandemic: Persistence of Prenatal SARS-CoV-2 Antibodies in Newborn Blood Spots
Source: Int J Neonatal Screen. 2023 Aug 2;9(3):43. doi: 10.3390/ijns9030043 (PMC10443380; doi:10.3390/ijns9030043)
Supplement: Supplementary file 1 [file IJNS-09-00043-s001.zip › IJNS-2496885-supplementary.pdf]

Supplemental Figures and Tables

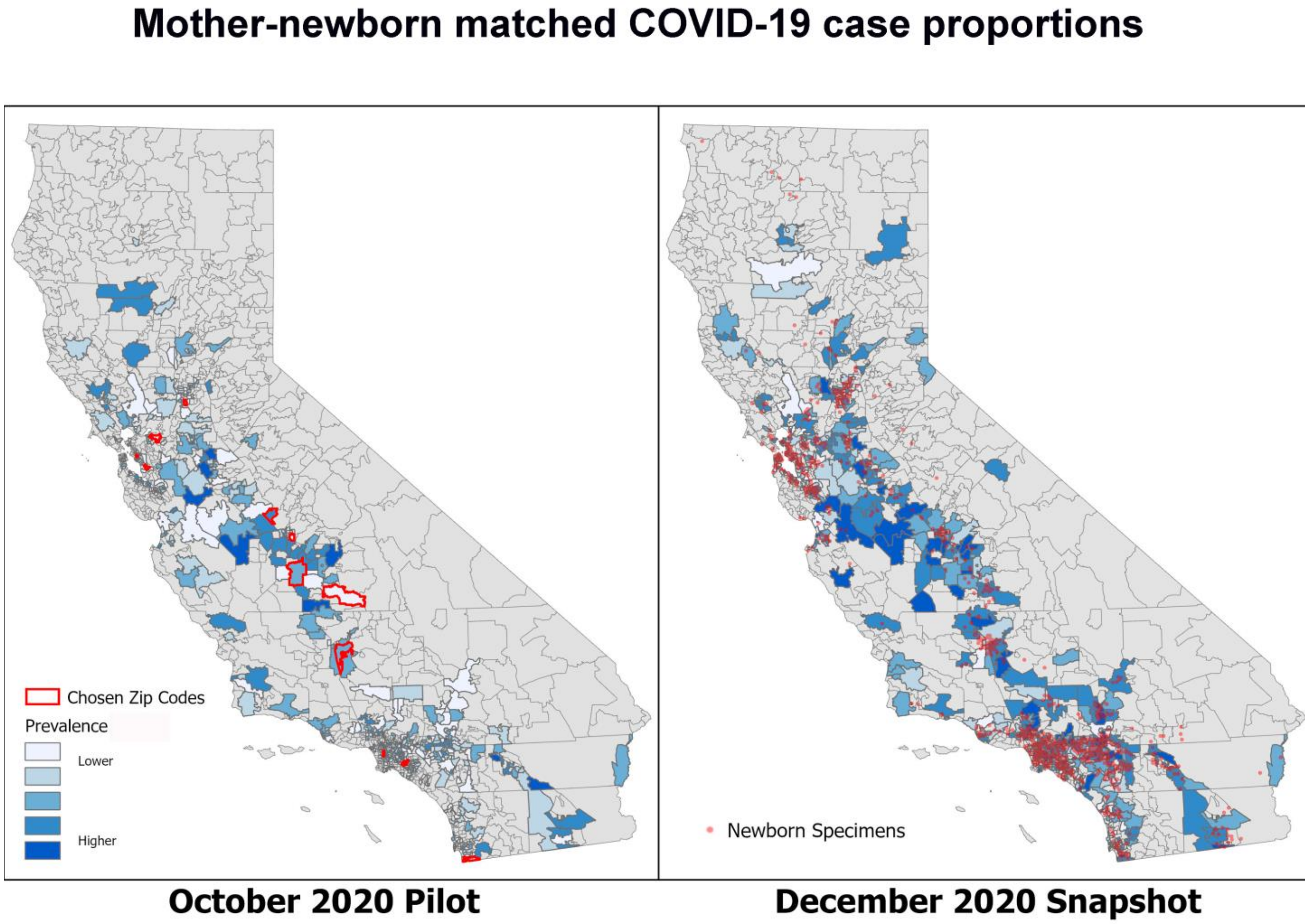

Figure S1. Prevalence proportions were calculated as the number of women who gave birth in a given month who had a CalREDIE laboratory confirmed case of COVID-19 divided by the number of women who gave birth in a zip code by month of birth. No results were shown in areas with fewer than eight births in a given month to reduce unstable estimates. The darker colors indicate higher proportions. Due to issues of confidentiality, we are not reporting specific proportions. For October 2020, we show outlines of the zip codes selected for testing. In December 2020, the red dots indicate our snapshot sample of all the newborns tested by newborn screening on a single day. In October 2020, the prevalence of the maternal-newborn CalREDIE matches was approximately 3% statewide vs. 5% in the chosen zip codes, in contrast to the 11.9% observed in newborn DBS specimens tested for antibodies.

Table S1. Newborn S1 antibodies detected and mother–newborn linked episode days before and after birth.

Antibodies detected?	Not linked	-360 to -20 days	-19 to -1 days	0 days (birth date)	1 to 110 days after birth	Total
**Yes**	284 (11.0%)	153 (94%)	5 (16%)	9 (43%)	2 (2%)	453 (15.7%)
**No**	2294 (89.0%)	10 (6%)	26 (84%)	12 (57%)	95 (98%)	2437 (84.3%)
**Total**	2578 (100.0%)	163 (100%)	31 (100%)	21 (100%)	97 (100%)	2890 (100.0%)
